# Safety of Antiretroviral Exposure During Pregnancy: Opportunities to Close Data Gaps

**DOI:** 10.1093/ofid/ofae423

**Published:** 2024-07-23

**Authors:** William R Short, Emily S Miller, Joanne Simone, Anne Statton, Sarah Finocchario-Kessler, Margaret Lampe

**Affiliations:** Department of Medicine, Division of Infectious Diseases, Perelman School of Medicine, University of Pennsylvania, Philadelphia, Pennsylvania, USA; Department of Obstetrics and Gynecology, Division of Maternal Fetal Medicine, Alpert Medical School of Brown University, Providence, Rhode Island, USA; Francois-Xavier Bagnoud Center, School of Nursing, Rutgers, The State University of New Jersey, Newark, New Jersey, USA; Mother and Child Alliance, Chicago, Illinois, USA; Department of Family Medicine & Community Health, University of Kansas Medical Center, Kansas City, Kansas, USA; Division of HIV Prevention, National Center for HV, Viral Hepatitis, STD and TB Prevention, Centers for Disease Control and Prevention, Atlanta, Georgia, USA

**Keywords:** antiretroviral therapy, birth defects, HIV, pregnancy, Pregnancy Registry

## Abstract

Pregnant persons with chronic health conditions often require pharmacotherapy to remain healthy. The Antiretroviral Pregnancy Registry is a prospective, international, voluntary, and exposure registry that collects information on antiretroviral (ARV) exposure; however, a minority of providers use the registry, leaving critical gaps to guide prescribing in this population. The Task Force for the Elimination of Perinatal HIV Transmission in the United States, funded by the Centers for Disease Control and Prevention, has identified the monitoring of ARV safety as a paramount concern in the ongoing mission to eliminate perinatal human immunodeficiency virus (HIV) transmission. As active members of this task force, we urge all healthcare providers who care for pregnant individuals to prioritize reporting all ARV exposures to the registry.

## Teaser

The Antiretroviral Pregnancy Registry is an international, voluntary registry that prospectively gathers data on antiretroviral exposure. Despite its importance, only a minority of providers use the registry, resulting in significant gaps in critical information needed to guide prescribing practices during pregnancy.

Pregnant persons with chronic health conditions almost always require pharmacotherapy to remain healthy [1]. This can present challenges because most medications undergo limited evaluation of effectiveness and safety during pregnancy. Consequently, clinicians are faced with the dilemma of either transitioning to older, often less efficacious drugs or continuing more contemporary medications initiated before pregnancy, despite limited data. This paradigm is particularly salient for antiretroviral (ARV) medications used for the treatment of human immunodeficiency virus (HIV) infection. ARVs initiated or continued during pregnancy not only maintain the health of the pregnant person but also dramatically reduce the risk of transmitting HIV to the fetus or newborn.

## STATEMENT OF THE PROBLEM

Three crucial evaluation pathways are essential for recommending the use of ARVs during pregnancy: assessing efficacy, conducting pharmacokinetic (PK) evaluations, and ensuring safety [2]. These are essential to ensure that HIV is adequately treated and the fetus is not exposed to a teratogenic agent. Pregnant persons are often not included in prelicensure studies of new drugs unless the drug is being studied for a pregnancy-related indication [3]. In the setting of HIV, some studies exclude cisgender women altogether, leaving enormous gaps in the data needed to safely provide care to pregnant persons living with HIV.

Typically, efficacy for HIV treatment is established through studies conducted in nonpregnant persons. Therefore, most drugs have data available once they are approved by the Food and Drug Administration. Furthermore, despite the long-standing knowledge of physiologic changes that occur in pregnancy, with resultant changes in drug absorption, distribution, metabolism, and excretion, PK data on ARVs in pregnancy remain limited. These clinical trials receive limited funding, leading to a median 6-year gap between ARV approval and the first published PK data in pregnancy [3].

One source of data on teratogenicity comes from animal studies. However, the predictive value for an effect on humans is unknown [4]. Data on outcomes after exposure to new ARV agents come from case reports, cohort studies, medical billing data, or registries. It is critical that we promptly collect prospective, nonbiased data on new ARV agents, so it does not take many years after the drug is approved by the Food and Drug Administration and widely used in nonpregnant persons to have adequate safety data to recommend or not recommend its use during pregnancy.

The Antiretroviral Pregnancy Registry (APR) is a prospective, international, exposure registry that was established in 1989 to collect information on ARV exposure. Healthcare providers voluntarily submit reports with the goal of detecting any major teratogenic effects involving any of the registry drugs to which pregnant persons are exposed. The information must be collected prospectively, meaning that the pregnant person (case) needs to be registered before the pregnancy outcome of the case is known [5].

The APR has predefined analytical methods and criteria for recognizing a potential signal. Researchers analyze data from the registry on birth outcomes for 200 infants who were exposed to an ARV drug during the first trimester of pregnancy. These data are viewed as sufficient to detect a doubling of the overall risk of major structural or chromosomal birth defects associated with that drug compared with the general population. The prevalence of major structural or chromosomal birth defects among live births in the general US population is 2.72%, as determined by the Metropolitan Atlanta Congenital Defect Program, a population-based tracking system for birth defects [5]. A cohort of 1000 is sufficient to detect a 1.5-fold increase in the risk of overall birth defects. For rare conditions such as neural tube defects (NTDs), which occur in <1 infant per 1000, a sample size of 2000 is required to detect a 3-fold increased risk [6].

**Figure 1.**
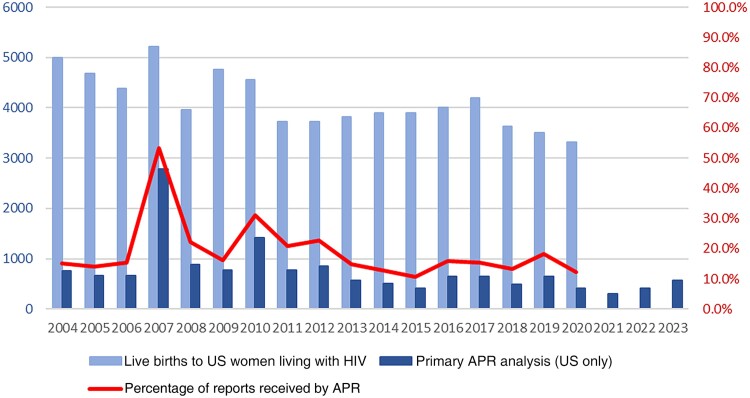
Prospective reports to the Antiretroviral Pregnancy Registry among live births to women with human immunodeficiency virus (HIV) in the United States. (Some people who do not identify as women may be included in the numbers.) Abbreviation: APR, Antiretroviral Pregnancy Registry.

There have been declining numbers of reports to the APR in recent years. Some contributing factors are that the onus of data entry is on individual providers, and they likely have no incentive beyond a personal commitment to supporting data for drug safety. Figure 1 highlights the number of prospective reports to the APR among live births to women with HIV in the United States. Evidence of this strain, and resultant gaps in the data available, are underscored by the finding that the APR receives a minority of reports compared with the number of pregnant persons living with HIV who give birth annually [5]. In the past, some clinicians have chosen to report birth defects only retrospectively. By not submitting all cases prospectively, there is no denominator to determine the percentage of defects occurring out of the total.

## IMPLICATIONS

The stories of efavirenz (EFV) and dolutegravir (DTG) provide historical perspectives on why reporting to the APR is critical. Early data from animal studies of EFV and retrospective case reports in humans [7, 8] raised concerns about the potential for congenital nervous system abnormalities and NTDs when EFV was taken around the time of conception and in early pregnancy. EFV was not recommended for use in women of reproductive potential or pregnant women, yet after approximately 10 years adequate data were available to rule out an association between EFV use and NTDs [9, 10]. Similarly, early data from an active surveillance study of birth defects in Botswana, including 426 preconception DTG exposures, suggested a possible association between NTDs and DTG use at conception [11]. However, data from expanded and ongoing surveillance of DTG use in Botswana found there was no detectable increase in NTDs or major external structural abnormalities among >11 000 exposures to DTG at conception captured in the Tsepamo Study from 2014 to 2022 [12].

A similar study in Eswatini also found no increase in NTDs with preconception DTG exposure [13]. In the United States, a cohort study using healthcare claims data did not find an increased risk of NTDs with use of DTG [14]. As reported, perinatal DTG exposures in the United States have increased, and the latest interim APR report included sufficient data to state that DTG is not associated with NTDs [5]. Such data can influence the polices developed to drive recommendations for countries all over the world. This change over time demonstrates the importance of reporting perinatal ARV exposures to the APR so that data are sufficient to draw timely conclusions.

New HIV medications are being developed each year, and gaps in evidence remain. For example, long-acting injectable cabotegravir is now widely used in clinical practice settings, thus possibly during periconception, a key time in fetal organogenesis. Minimal data have been reported on cabotegravir exposure during pregnancy, which serves as a call to action for HIV providers.

## RECOMMENDATIONS

The Task Force for the Elimination of Perinatal HIV Transmission in the United States, convened by CityMatCH with support and involvement of the Centers for Disease Control and Prevention, has identified safety monitoring of ARVs during pregnancy as a paramount concern in the mission to eliminate perinatal HIV transmission. (CityMatCH is a national membership organization of city and county health departments’ maternal and child health; programs and leaders representing urban communities in the United States; its mission is to strengthen public health leaders and organizations to promote equity and improve the health of urban women, families, and communities [15]).

As active members of this task force, we are reaching out to urge all healthcare providers who care for pregnant individuals to prioritize reporting all ARV exposures to the APR (at https://www.apregistry.com/). Reporting is also recommended by the Department of Health and Human Service Panel on Treatment of HIV During Pregnancy and Prevention of Perinatal Transmission. We also advocate for the APR to incentivize reporting practices to mitigate further delays in reaching the numbers of reports needed to thoroughly evaluate the risk of birth defects, such as providing reimbursement to reporters and empowering more members of the care team to report.

Artificial intelligence (AI) can significantly streamline the process of extracting data from medical charts and reporting it to the APR. Using natural language processing and optical character recognition, AI can efficiently interpret and convert unstructured text and handwritten documents into standardized, machine-readable data. This data can then be integrated seamlessly with electronic health record systems. AI automates data entry, ensuring accuracy and reducing manual errors while also enabling real-time reporting. Leaders in the fields of HIV and maternal and child health should unite to establish a shared approach to improve reporting to the APR and consider new efficient approaches to ensuring medication safety in pregnancy.
